# Enhanced recovery protocol in laparoscopic liver surgery

**DOI:** 10.1007/s00464-020-07470-2

**Published:** 2020-02-27

**Authors:** Johanna Savikko, Leena Vikatmaa, Anna-Maria Hiltunen, Noora Mallat, Eija Tukiainen, Sari-Mari Salonen, Arno Nordin

**Affiliations:** 1 Department of Transplantation and Liver Surgery, HUS Helsinki University Hospital, Haartmaninkatu 4, P.O. Box 340, 00029 Helsinki, Finland; 2 Department of Anesthesiology and Intensive Care Medicine, HUS Helsinki University Hospital, Helsinki, Finland; 3 Department of Industrial Engineering and Management, HEMA Institute, Aalto University, Helsinki, Finland; 4 Nordic Healthcare Group, Helsinki, Finland

**Keywords:** Laparoscopic liver surgery, Enhanced recovery, Opioid-sparing pain management

## Abstract

**Introduction:**

Enhanced recovery protocols (ERP) accelerate recovery and shorten postoperative hospital stay. This increased knowledge of ERPs has also gradually implemented into liver surgery. However, in laparoscopic liver surgery (LLS), the experience of optimized perioperative care protocols is still limited.

**Methods:**

We prospectively studied the implementation of multimodal ERP principles to LLS in the first 100 consecutive patients. Opioid-sparing multimodal pain management was applied together with early mobilization already in the postoperative care unit (PACU). Drains and catheters were avoided and per oral intake was initiated promptly. Primary pain control was achieved with iv NSAIDS, low-dose opioid and corticosteroids. Combination of per oral ibuprofen and long-acting tramadol was routinely administered shortly after operation. The multiprofessional adherence to the protocol was also evaluated.

**Results:**

Investigated LLS was performed during Aug 2016–Apr 2019. Operations were done due to malignancy in 83 (83%) of cases, mostly for colorectal liver metastases (*n* = 52, 52%). Forty-eight (48%) of the operated patients were female. Median age was 65 years (range 17–91). The American Society of Anaesthesiologists Physical Status (ASA) classification median was three. Median postoperative hospital stay was 2 days (range 1–8 days). More than seventy percent of patients were discharged by the second postoperative day and nearly ninety percent by the third postoperative day. Complications after surgery were few. The new ERP elements were adopted in most of the cases.

**Conclusions:**

ERP was introduced safely and effectively after LLS. The adherence to the ERP was good. Routine discharge 1–2 days after LLS is realistic and achievable.

Laparoscopic liver surgery (LLS) has evolved over the past two decades and is today a widely accepted standard of practice [1, 2]. Recently it has been shown in randomized controlled trials that LLS is associated with significantly less postoperative complications and pain than open surgery [3, 4]. In addition, LLS also showed superior cost-effectivity [3].

Enhanced recovery protocols (ERP) accelerate patient’s recovery and shorten hospital stay by the optimization of whole perioperative care. Together with improved laparoscopic techniques ERP protocols have progressively decreased the need for hospitalization in many abdominal operations. Although some recent studies have indicated that minor laparoscopic liver resection may be performed even as a day-case surgery in highly selected patients [5, 6], the experience of optimized ERP is still limited in LLS. Clear superiority of ERP over standard protocols in various abdominal operations make well powered randomized controlled trials (RCT) ethically questionable also for LLS. In addition, recently it has turned out that it is challenging to recruit enough liver surgery patients even within multicentre studies for double-blind RCT [7].

ERP has been a standard practice in our liver surgery unit since 2013 and patients treated according to the protocol have been very satisfied. The hospitalization has shortened by 2 days. Mean length of hospital stay (LOS) was 4 days including major liver surgery, but there was no difference in LOS between LLS and open liver surgery [8]. Since then we have improved our protocol and adjusted it to better support the recovery of laparoscopically operated patients. At the same time, the laparoscopic techniques in liver surgery have evolved in our unit and the number of laparoscopic liver resections has increased markedly.

The aim of this study was to investigate the effect of implementation of a modified ERP in LLS on the length of postoperative hospital stay, morbidity and adherence to the protocol.

## Methods

### Study design and patient selection

From August 2016, ERP was implemented for patients that underwent LLS at the Transplantation and Liver Surgery Unit of HUS Helsinki University Hospital, a high-volume hepatobiliary unit acting as a tertiary centre for entire Finland (population 5.5 Mio. people). Since 1982, more than 3000 liver operations have been performed in our unit in addition to more than 1300 liver transplantations. The annual volume is approximately 200 liver resections, mostly for liver metastases of colorectal cancer. The five-year survival of these colorectal cancer patients after liver surgery is more than 60%^*9*^*.* The LLS training is executed according to the Southampton guidelines [9]. Currently, there are three surgeons capable to perform LLS. During 2016–18, approximately 20% of all liver surgery was performed laparoscopically.

All 23 enhanced recovery elements defined in recent ERAS Society recommendations for liver surgery were taken into account in our protocol [10]. The items used in our protocol are presented in Table 1 and the new elements are highlighted compared to our previous protocol. All patients who underwent LLS between Aug 18, 2016 and Apr 23, 2019 were included. Patients were introduced to the protocol by the surgeon at the preoperative visit, and they also received written information and were educated by nurses.

**Table 1:** The comparison of enhanced recovery elements in our previous and new protocol. The added elements are shown in italics

New protocol	Previous protocol
Preop. physiological optimization	Preop. physiological optimization
Avoid preop. bowel preparation	Avoid preop. bowel preparation
Preop. fasting + carbohydrate drink up to 2 h before surgery	Preop. fasting + carbohydrate drink up to 2 h before surgery
*Avoid anaesthetic premedication*	
Prophylaxis against thromboembolism	Prophylaxis against thromboembolism
*Patients walked from the ward to the operation room together with a nurse*	Antimicrobial prohylaxis
Antimicrobial prohylaxis	Standard anaesthetic protocol
*Perioperative steroid administration*	Postop. nausea and vomiting—multimodal approach
Standard anaesthetic protocol	Avoid nasogastric tube
Postop. nausea and vomiting—multimodal approach	Prevent intraop. Hypothermia
Avoid nasogastric tube	Periop.fluid management—goal-directed fluid therapy
Prevent intraop. Hypothermia	Avoid routine surgical drainage
Periop.fluid management—goal-directed fluid therapy	Urinary drainage: 1–2 days only
Avoid routine surgical drainage	Prevention of ileus—multimodal approach
*Mobilization already in PACU unit*	Postop. analgesia—thoracic epidural/wound catheter (avoid opiates)
*Urinary drainage removed already in operating room*	Periop. nutritional care (supplements)
Prevention of ileus—multimodal approach	Postop. glucose control
Postop. analgesia—thoracic epidural/wound catheter (avoid opiates)	Early mobilization—intensive physiotherapy (twice daily)
Periop. nutritional care (supplements)	
Postop. glucose control	
Early mobilization—intensive physiotherapy (twice daily)	

There was no need for institutional review board permission for this prospective study. All patients having laparoscopic liver surgery were treated according to it and no additional elements were used compared to the standard care. No personal data can be identified in the report of the study.

### Surgical technique and perioperative management

Liver resections were graded according to the Iwate scoring system specifically designed for LLS [11]. Liver resections were also categorized as minor (fewer than three segments, including multiple non-anatomical resections), or major resection (three or more segments). Three surgeons with expertise in both laparoscopic and open liver surgery performed all procedures. The aim of the surgical treatment was to achieve complete macroscopic resection of the hepatic lesion(s) in all cases. Intraoperative ultrasound with the optional use of contrast media was systematically performed to confirm the number and size of the lesions, to define their relationship to the major intrahepatic vascular structures and to look for occult liver metastases. All port sites were injected with 7.5 mg/ml ropivacaine either at the start or towards the end of the procedure. The decision to use drainage was left to the surgeon but the unit’s general policy was to avoid drains. Drains were generally removed on the first postoperative day (POD). Urinary catheter was removed at the end of the operation unless a specific reason to leave it existed.

All patients received low-molecular-weight heparin (LMWH) enoxaparin sodium 40 mg subcutaneously in the evening before the operation. All patients received a high-rich carbohydrate recovery drink (Nutricia preOp, Nutricia Medical Ltd. Turku, Finland) two hours before the operation. Premedication was not included in the protocol, but pregabalin 75–150 mg could be given when considered necessary. Patients walked from the ward to the operation room together with a nurse. Anaesthesia was induced with propofol, fentanyl and rocuronium. Dexamethasone 10 mg iv for the prevention of postoperative nausea and vomiting was administered at the induction of anaesthesia [12]. Intraoperatively, anaesthesia was maintained with desflurane and fentanyl boluses or alfentanil infusion. Rocuronium boluses were used for muscle relaxation, and either neostigmine-glycopyrronium or sugammadex was used for the reversal of relaxation. Bolus doses of ketorolac 30 mg iv, ketamine 2.5–5 mg iv and oxycodone 3–4 mg iv were administered towards the end of anaesthesia. Central venous catheter was asserted only if the peripheral veins were problematic. Central venous pressure measurements were considered unreliable in the laparoscopic setting with steep reverse Trendelenburg position and laparoscopy-induced intraperitoneal pressure. The control of blood loss was based on good surgical technique, limited fluid administration (0.5–1 ml/kg/h) and continuous assessment of individual physiological parameters, such as arterial blood pressure, pulse pressure variation, sufficient diuresis and acid–base homeostasis. Transfusion trigger was individually set both intra- and postoperatively at haemoglobin 80–90 g/l according to the patient’s comorbidities.

Patients were discharged to the ward after 3–4 h follow-up in the postoperative care unit (PACU). Mobilization and positive expiratory pressure exercises were started already in the PACU and patients were encouraged to walk to the toilet assisted by a nurse. All patients were allowed fluids approximately 3–4 h after the procedure and food on the evening of the operation day. In the evening they also received a second high-rich carbohydrate recovery drink. Laxatives (mainly lactulose) were used routinely from the day of surgery onwards. A once daily subcutaneous injection of LMWH was maintained during the hospital stay. In case of malignant tumours, LMWH was continued for three weeks.

Multimodal opioid-sparing pain management was applied during the entire hospital stay [13]. Postoperative pain was regularly assessed with either visual analogue scale (VAS) or verbal rating scale (VRS). Ibuprofen (400–800 mg three times daily) was started in the evening of the operation. If NSAIDs were contraindicated, acetaminophen (500–1000 mg three times daily) was used instead. Extended release tramadol (75–150 mg twice daily) was also added to the medication in the evening of operation. If needed for severe pain, the patient could also receive oral oxycodone (0.1–0.2 mg/kg). At discharge, ibuprofen and long-acting tramadol were prescribed and advised to be used at home for pain relief for several days.

### Discharge and follow-up

Criteria for discharge were sufficient pain control by oral analgesics, independent mobilization, eating and drinking without problems and no untreated surgical complications. The goal of the ERP program was to achieve discharge at POD 1–3 after a laparoscopic liver resection. Complications were graded according to the Dindo-Clavien classification [14].

Patients were contacted by phone 3 days after discharge by a ward nurse. During the follow-up call, the nurse filled out a structured questionnaire on patients’ pain, mobilization, bowel function, overall well-being and experience of care. Patients pain level was assessed with three-point scale: no pain—moderate pain—severe pain. Thereafter, they were asked whether the pain was managed sufficiently, moderately or poorly. The satisfaction towards care overall was asked on a four-point scale: excellent—good—fair—poor. The aim of the phone call was to assure a safe transition from hospital to home and track possible complications as early as possible.

### Adherence to protocol

One aim of this study was to examine the multimodal adherence to the protocol. The investigated parameters included medication and nursing-related parameters. This data were collected from medical records.

## Results

### Study population

All hundred patients having LLS during the study period were included. Indications for liver resections and detailed demographic data are presented in Table 2. Fifty-two (52% of cases), operations were done due to colorectal livers metastases, 16 (16%) hepatocellular carcinoma, five peripheral cholangiocarcinoma (5%), 10 other liver metastases (10%) and the remaining 17 (17%) of the cases for benign liver tumours. Other liver metastases were metastases of melanoma, gastrointestinal stromal tumour, neuroendocrine tumour, mammary carcinoma or thyreoid carcinoma. Benign tumours included adenoma, hamartoma, cystadenoma, focal nodular hyperplasia, endometriosis and hemangioendothelioma. Forty-eight (48%) of the operated patients were female. Median age was 65 years (range 17–91). The ASA classification median was three. Sixteen patients (16%) of the cohort had the diagnosis of liver cirrhosis; fifteen patients treated for hepatocellular carcinoma and one with cholangiocarcinoma. All these patients were in compensation with their liver disease. The median Child Pugh score was 5 (range 5–7) and class was A for thirteen of these patients, two had class B cirrhosis.

**Table 2:** Indications for liver resections and detailed demographic data

	N	Median	Range (IQR)
Age (years)		65	17–91 (57–71)
Gender (F: M)	48: 52 (48: 52)		
Length of postoperative			
hospital stay		2	1–8 (2–3)
ASA classification			
I	6 (6)		
II	34 (34)		
III	53 (53)		
IV	7 (7)		
Histology			
CRLM	52 (52)		
HCC	16 (16)		
CCA	5 (5)		
Other metastases	10 (10)		
Benign tumour	17 (17)		

Values in parentheses are percentages unless indicated otherwise

*IQR* interquartile range; *ASA classification* The American Society of Anaesthesiologists Physical Status; *CRLM* colorectal liver metastases; *HCC* hepatocellular carcinoma; *CCA* cholangiocarcinoma

### Surgical technique and perioperative details

Details of surgical categories and perioperative details are shown in Table 3. Most of the operations (*N* = 91) were minor liver resections having fewer than three segments, including multiple non-anatomical resections. Nine major liver resections were right hemihepatectomies. The mean Iwate score describing the difficulty of LSS was 4.4, and the median 4 with range 2–10. The number of patients having simultaneous, multiple non-anatomical resections was 10 (10%). Three patients (3%) had had a previous open liver resection due to colorectal metastases. The intraoperative use of laparoscopic ultrasound did not alter markedly the flow of the operation and had only marginal if any effect on blood loss during the operation. Median blood loss was 100 ml and only one patient received blood transfusion.

**Table 3:** Details of surgical categories and perioperative details

	N	%	Median	Mean	Range
Operation category					
Minor operation	91	91			
Major operation	9	9			
Iwate score			4	4.4	2–10
Blood loss (ml)			100	208	0–1600
Operation time (min)			131	141	46–334

### Adherence to the ERP elements

Compliance to the elements in the protocol is demonstrated in Table 4. All except one patient (99%) received the preoperative drink two hours before induction of anaesthesia. Only five (5%) patients received pregabalin as a premedication before the operation. This allowed the majority of patients to walk from the ward to the operation theatre accompanied by a nurse. Altogether 91 (91%) of the patients received dexamethasone during the anaesthesia induction and 84 (84%) patients received ketorolac, ketamine and oxycodone in the end of the anaesthesia. All patients received local anaesthesia to the port sites during the operation. The urine catheter was removed in the PACU from 72 (72%) patients. Two thirds of the patients were successfully mobilized during recovery in the PACU.

**Table 4:** Adherence to the enhanced recovery protocol

Protocol component	N (%) of patients or mean (SD)
Preoperative drink	99 (99%)
No premedication	95 (95%)
Dexamethasone 10 mg iv during anaesthesia induction	91 (91%)
Ketorolac 30 mg, ketamine 2.5–5 mg and oxycodone 3–4 mg iv at the end of surgery	84 (84%)
Port sites infiltrated with ropivacaine 7.5 mg/ml	100 (100%)
Urine catheter removed in PACU at the latest	72 (72%)
Mobilization in the PACU: sitting and/or walking	66 (66%)
Recovery time in the PACU^a^	243 min (73)

*PACU* postanaesthesia care unit

^a^Time from the arrival to the PACU until the discharge criteria were met

### Hospital length of stay

**Fig. 1:**
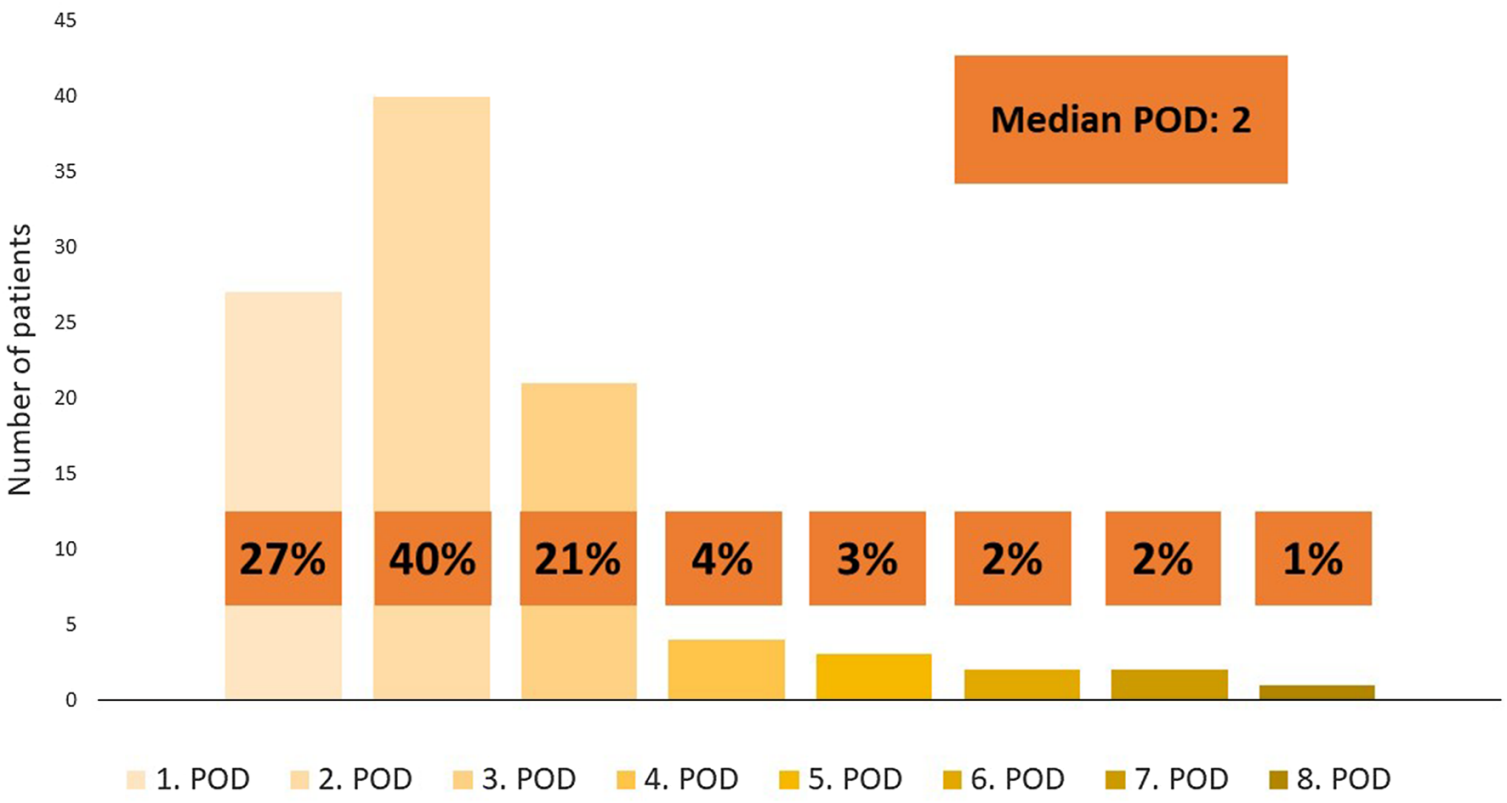
Postoperative length of hospital stay is shown here. Eighty-eight (88%) patients were discharged by the 3rd postoperative day. Most of the patients were discharged at the 2nd postoperative day

Hospital LOS is shown in Fig. 1. The median postoperative hospital stay was 2 (range 1–8) days. Eighty-eight (88%) patients were discharged by the third postoperative day. Of the ninety-one patients who underwent minor LLS 27 (30%) were discharged at POD 1, 38 (42%) at POD 2 and 19 (21%) at POD 3. Nine patients who underwent major LLS stayed somewhat longer at hospital; 2 (22%) were discharged at POD 2, 2 (22%) at POD 3, 3 (33%) at POD 4, 1 (11%) at POD 5 and 1 (11%) at POD 6. There was no difference in median hospital LOS if the patient had cirrhosis diagnosis or not. The median hospital LOS in the group of cirrhotic patients was 2 (range 1–7) days.

### Postoperative pain management

Oxycodone was administered at the ward to 36 (36%) patients during the day of the operation (median per recipient 10 mg), to 42 (42%) during POD 1 (11 mg) and to 15 (15%) during POD 2 (10 mg). Altogether 38 (38%) patients did not receive any oxycodone during their stay on the surgical ward. Tramadol was dosed regularly to 85 (85%) of patients and NSAID to 70 (70%) of patients at the surgical ward.

**Fig. 2:**
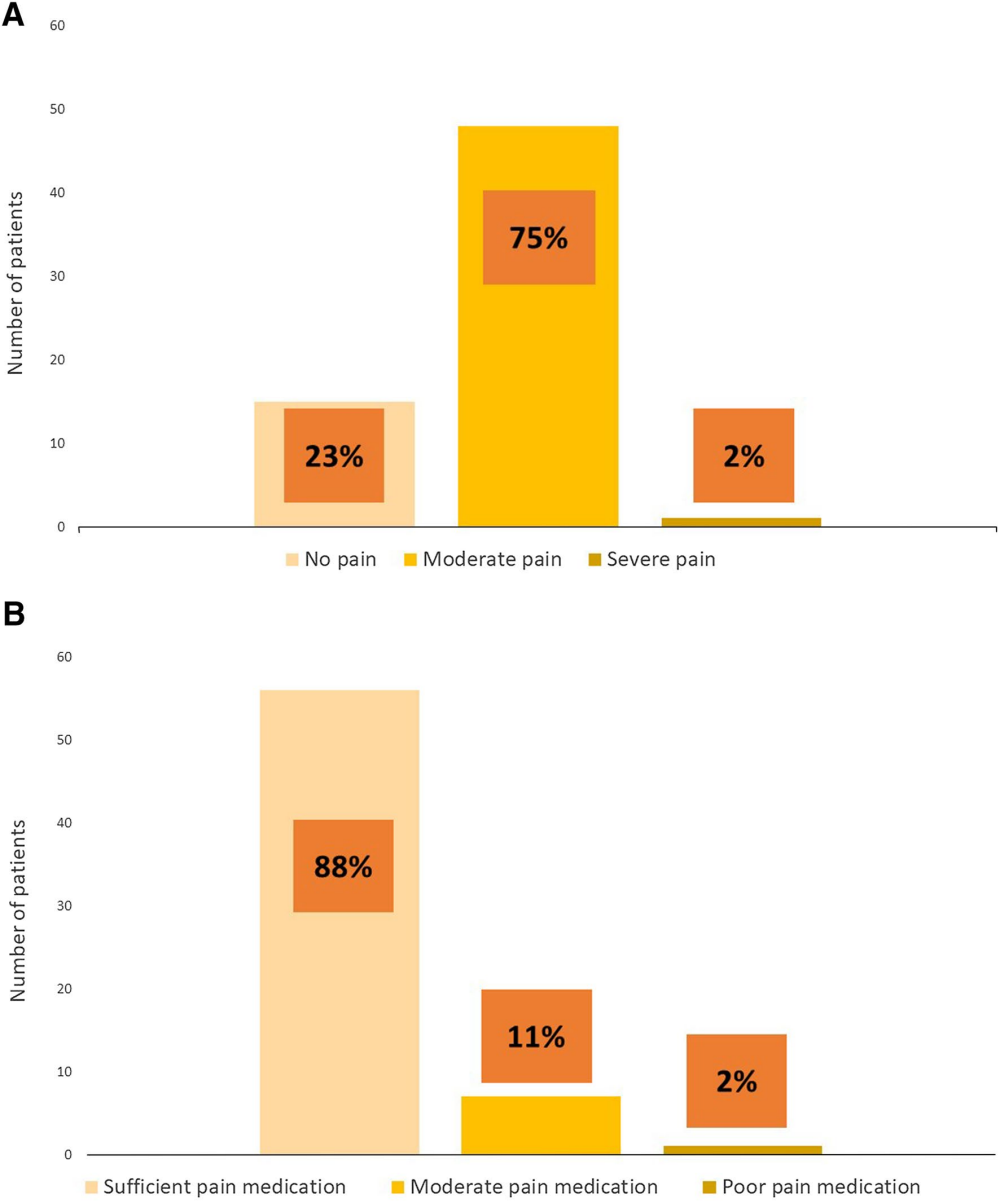
Pain level and pain medication sufficiency three days after discharge are presented here. The majority of the patients experienced moderate pain (**A**), which is common after abdominal surgery. However, most of the patients felt that their pain medication was at a sufficient level (**B**)

Pain level and pain medication sufficiency three days after discharge are presented in Fig. 2a, b. In total, sixty-four patients (64%) responded the questions of pain and pain medication sufficiency three days after discharge. Three days after discharge, fifteen (23%) of these patients reported no pain at all, fourty-eight (75%) patients experienced moderate pain and one patient had severe pain. When the efficiency of pain medication was asked, the majority (*N* = 56, 88%) of these patients felt that the medication was sufficient. Seven patients (11%) had tolerable pain medication. Only one patient (2%) reported poor pain medication. However, the patient who experienced severe pain expressed his pain medication was sufficient.

### Complications

Complications were rare, and morbidity was low after LLS with this ERP. Ninety-three patients had no complications according to the Dindo-Clavien classification, three patients had grade II complication, two grade IIIa complication and two IIIb complication. Only one patient with liver cirrhosis had a IIIb complication; a haemorrhage at the evening of the operation, which was easily managed laparoscopically.

Fluid collection was punctuated from two patients subcutaneously, one patient was endoscopically treated with a stent due to a biliary obstruction and two patients were re-operated; one due to an intestinal lesion during laparoscopy and one for haemorrhage. Two patients (2%) were re-admitted to the liver surgery ward. Both of them were male and they were discharged at day one after operation, but they had to come back to hospital because of bowel dysfunction. At hospital, they were treated conservatively, and were discharged again after a couple of days of hospitalization. The thirty-day and ninety-day mortality were zero.

### Patient satisfaction

**Fig. 3:**
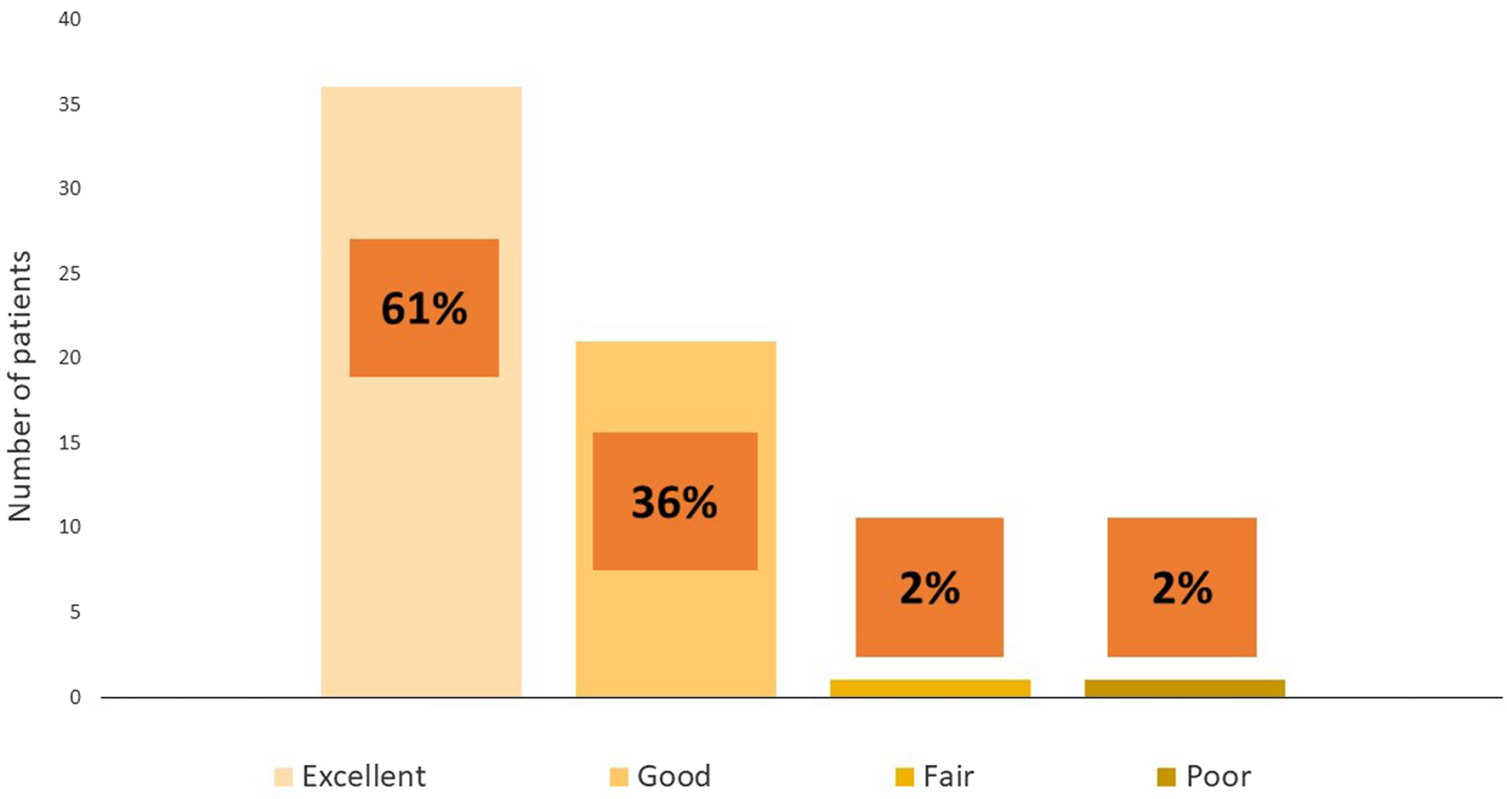
Patient satisfaction 3 days after discharge is shown here. Most of the patients described their experience either as “Excellent” or “Good”

Patient satisfaction 3 days after discharge is shown in Fig. 3. In total, fifty-nine (59%) patients responded the questions of satisfaction three days after discharge. Of the patients who answered the question most (*N* = 57, 97%) described their experience either as “Excellent” or “Good”. In one case only the patient’s spouse was reached via telephone because the patient was busy with his normal daily activities.

## Discussion

Laparoscopic liver surgery is a modern treatment of choice for most of liver tumours. Enhanced recovery protocols have shortened length of hospital stay and improved outcomes in various types of abdominal surgery including liver surgery. However, the exact variables and factors affecting the better results and the adherence to the protocols are still largely unknown. Here we describe in detail an enhanced recovery protocol in laparoscopic liver surgery. The adherence to protocol was also investigated.

Somewhat modified ERPs have been successfully utilized in our clinic since 2013. The original protocol was designed for open liver surgery and at that time the part of LLS was quite small. Previously we could see no difference in postoperative recovery and LOS stay between open and laparoscopic liver surgery; although the median LOS was only 4 days including also major liver surgery [8]. This suggested that our protocol was not optimal for laparoscopically operated patients. In last two to three years, the amount of laparoscopic liver surgery has increased in our unit. That in mind we designed a new ERP for LLS. More attention was paid to the patient mobilization in both pre- and postoperative phases. Other new elements introduced into this protocol were: no premedication was given, patients walked to the operating room, and the mobilization was started already in the recovery room. In addition, some modification to medication was done. During anaesthesia routine use of cortisone in induction and NSAIDs at the end of the operation were started. In the recovery phase, use of opioids was kept as low as possible and postoperative pain was mainly treated with ketamine, metamizole and long-acting tramadol.

According to our current results, routine discharge one to two days after LLS is possible and safe. To the best of our knowledge, this is the largest cohort of LLS patients showing this rapid recovery with very few complications. In our study, the median of LOS was 2 days including also patients having major liver surgery or cirrhosis as an underlying liver disease. Complications were rare and readmission rate was very low, less than 2%. With this new protocol the median LOS was shortened by 2 days compared to our previous study. Similar findings have been shown by de’Angelis and Tranchart, but in their studies patients have been highly selected according to age and comorbidities [5, 6]. Our patient material was similar to the one we have reported earlier. The fact that most patients were operated for malignant tumours, median age was 65 years and median ASA class 3 underline this. Both French investigators have suggested that liver surgery can be performed even as day-case surgery in selected patients [5, 6]. Our current experience and results support also this kind of management of patients after LLS. However, in a long-distance country like Finland, we have had no special need to this.

The shorter the LOS the better patients recover from surgery. This is important as a significant proportion of the LLS is performed due to colorectal liver metastases. Adjuvant therapy after surgery is commonly administered. Thus, improved recovery should be beneficial also for the overall oncological results. Interestingly, enhanced recovery protocols may have a role in improved cancer outcomes owing to changes in a cell-mediated immunity and also because patients may receive earlier postoperative adjuvant therapy [15, 16].

In the studies of ERP in LLS the knowledge of adherence to the protocols is mostly missing [10]. In our cohort, the compliance to the protocol was good and the new elements were adopted in most of the cases. However, missing data were somewhat a problem and we suggest that specific notes describing the fulfilled elements should be taken. Electronic notes in a checklist form would probably also make the protocols more effective. The elements acquiring the activity of ward nurses were materialized in nearly all cases. However, actions needed in PACU were not always fulfilled. This may depend on both improper education and missing resources in these busy units. There is a continuous need for multimodal staff education to get the adherence better. This may be challenging in recovery room units treating various types of patients in many surgical specialties, like ours at Helsinki University Hospital. We believe that by achieving early mobilization and other significant elements more completely fulfilled we still could shift more patients to be discharged at the first postoperative day.

Patients were commonly very satisfied with their treatment and based on discussions with the patients LLS was considered easier than they had expected. The follow-up call was performed and documented clearly only in 64% of the cases. Most likely the major reason for this is that patients were so healthy at the time of discharge that the follow-up call was missed and not scheduled according to the protocol. However, more thorough description of patients’ care experience is needed to understand better the patients’ views of how to improve the protocol. Moreover, as ERP is greatly collaboration between the patient and health care professionals, improved patient education is still needed to further develop the ERPs. This could be achieved by standardized patient information such as patient education videos or mobile applications supporting the pre- and postoperative phases of LLS.

Taken together our results demonstrate that ERP can be introduced safely and effectively after LLS. Routine discharge 1–2 days after LLS is realistic and achievable. Although the overall adherence to the protocol was quite high, we believe that even better results could be obtained by continuous multimodal staff information and improved patient education.

## References

[1] Abu Hilal M, Aldrighetti L, Dagher I, Edwin B, Troisi RI (2018). The Southampton consensus guidelines for laparoscopic liver surgery. From indication to implementation Ann Surg.

[2] Berardi G, Van Cleven S, Fretland ÅA, Barkhatov L, Halls M, Cipriani F, Aldrighetti L, Abu Hilal M, Edwin B, Troisi RI (2017). Evolution of laparoscopic liver surgery from innovation to implementation to mastery: perioperative and oncologic outcomes of 2238 patients from 4 European specialized centers. J Am Coll Surg.

[3] Fretland ÅA, Dagenborg VJ, Bjornelv GMW, Kazaryan AM, Kristiansen R (2018). Laparoscopic versus open resection for colorectal liver metastases. The OSLO-COMET randomized controlled trial. Ann Surg.

[4] Liang X, Ying H, Wang H, Xu H, Liu M (2018). Enhanced recovery care versus traditional care after laparoscopic liver resections: a randomized controlled trial. Surg Endosc.

[5] de’Angelis N, Menahem B, Compagnon P, Merle JC, Brunetti F, Luciani A, Cherqui D, Laurent A (2017). Minor laparoscopic liver resection: toward 1-day surgery?. Surg Endosc.

[6] Tranchart H, Fuks D, Lainas P, Gaillard M, Dagher I, Gayet B (2017). Laparoscopic liver surgery: towards a day-case management. Surg Endosc.

[7] Wong-Lun-Hing EM, van Dam RM, van Breukelen GJP, Tanis PJ, Ratti F (2017). on behalf of the ORANGE II collaborative group. Randomized clinical trial of open versus laparoscopic left lateral hepatic sectionectomy within an enhanced recovery after surgery programme (ORANGE II study). Br J Surg.

[8] Savikko J, Ilmakunnas M, Mäkisalo H, Nordin A, Isoniemi H (2015). Enhanced recovery protocol after liver resection. Br J Surg.

[9] Reijonen P, Kivelä A, Rantonen J, Juuti A, Salo J, Isoniemi H, Räsänen J, Nordin A (2019). Long-term outcome after sequential liver and lung metastasectomy is comparable to outcome of isolated liver or lung metastasectomy in colorectal carcinoma. Surg Oncol.

[10] Melloul E, Hubner M, Scott M, Snowden C, Prentis J, Dejong CH (2016). Guidelines for perioperative care for liver surgery: enhanced recovery after surgery (ERAS) society recommendations. World J Surg.

[11] Ban D, Tanabe M, Ito H, Otsuka Y, Nitta H (2014). A novel difficulty system for laparoscopic resection. J Hepatobiliary Pancreat Sci.

[12] Gan TJ, Diemunsch P, Habib AS, Kovac A, Kranke P (2014). Consensus guidelines for the management of postoperative nausea and vomiting. Anesth Analg.

[13] Kumar K, Kirksey MA, Duong S, Wu CL (2017). A review of opioid-sparing modalities in perioperative pain management: Methods to decrease opioid use postoperatively. Anesth Analg.

[14] Dindo D, Demartines N, Clavien P-A (2004). Classification of surgical complications. A new proposal with evaluation in a cohort of 6336 patients and results of a survey. Ann Surg.

[15] Fawcet WJ, Mythen MG, Scott MJP (2012). Enhanced recovery: more than just reducing length of stay?. Brit J Anaesth.

[16] Khuri SF, Henderson WG, DePalma RG, Mosca C, Healey NA, Kumbhani DJ (2005). Determinants of long-term survival after major surgery and the adverse effect of postoperative complications. Ann Surg.

